# Combining difference and equivalence test results in spatial maps

**DOI:** 10.1186/1476-072X-10-3

**Published:** 2011-01-10

**Authors:** Thomas Waldhoer, Harald Heinzl

**Affiliations:** 1 Department of Epidemiology, Center for Public Health, Medical University of Vienna, Borschkegasse 8a, A-1090 Vienna, Austria; 2 Center for Medical Statistics, Informatics, and Intelligent Systems, Medical University of Vienna, Spitalgasse 23, A-1090 Vienna, Austria

## Abstract

**Background:**

Regionally partitioned health indicator values are commonly presented in choropleth maps. Policymakers and health authorities use them among others for health reporting, demand planning and quality assessment. Quite often there are concerns whether the health situation in certain areas can be considered different or equivalent to a reference value.

**Results:**

Highlighting statistically significant areas enables the statement that these areas differ from the reference value. However, this approach does not allow conclusions which areas are sufficiently close to the reference value, although these are crucial for health policy making as well. In order to overcome this weakness a combined integration of statistical difference and equivalence tests into choropleth maps is suggested and the approach is exemplified with health data of Austrian newborns.

**Conclusions:**

The suggested method will improve the interpretability of choropleth maps for policymakers and health authorities.

## Background

A choropleth map consists of coloured or patterned areas which represent different values or categories of a quantitative attribute. Displaying health information data in choropleth maps has become common practice in spatial epidemiology.

Statistically significant deviations of the depicted values from a reference value are often highlighted in such maps. Their results, however, may lead to unwanted concerns and bewilderment in certain significantly worse regions. Inhabitants of those regions may put political pressure on local authorities and governmental agencies. However, in spatial units with many observed events, even tiny and irrelevant effects may show statistical significance. On the other hand, statistical difference tests usually have little statistical power for areas exhibiting few events which could intuitively lead to the false impression that areas with non-significant test results are close to the reference value.

Equivalence tests can provide useful information in addition to difference tests as the former require the specification of a conclusively substantiated equivalence range. We suggest the combined use of both difference and equivalence tests in spatial maps. We exemplify that this combined approach provides more insight into spatial conditions than sole difference tests. We think that it can considerably enhance the illustrative capability of choropleth maps in public health and epidemiology.

The paper is organised as follows. Basic ideas, statistical methods, and the combined approach are presented before the combined approach is applied to two data sets. A discussion and conclusions section closes the paper.

## Methods

The main features of difference and equivalence tests are motivated with the one-sample t-test. Based on it, the combination of both test principles is thoroughly ventilated. Although these considerations are general by nature, the application of permutation tests may pose additional questions which are considered and exemplified with standard mortality ratios (SMR's). Multiple testing and a Bayesian alternative approach are briefly considered as well.

### The one-sample t-test as difference test

Consider a single group with normally distributed outcomes, *X *~ N(μ,σ^2^), where μ and σ^2 ^are the unknown population mean and variance. The research question whether the population mean *differs *from a chosen constant *c *is usually answered with a one-sample t-test of the null hypothesis H_0_: μ = *c *on a prespecified significance level α. The corresponding non-directional two-sided alternative hypothesis is denoted by H_A_: μ ≠ *c*.

The null hypothesis H_0_: μ = *c *can be considered as intersection of two one-sided null hypotheses H_01_: μ ≤ *c *and H_02_: μ ≥ *c*, respectively. Testing them can be understood as a closed testing procedure which holds the multiple level α and a confirmatory directional conclusion is possible (see e.g. [1], [2]). The corresponding one-sided directional alternatives are H_A1_: μ >*c *and H_A2_: μ <*c*, respectively.

In the case of one-sample t-test the application of a two-sided level α test comprises the computation of a realisation *t *of a test statistic *T*, and its comparison with some lower and upper critical values *crit*_*low *_and *crit*_*upp*_, respectively. If *t *∉ [*crit*_*low,*_*crit*_*upp*_], then H_0 _will be rejected for the non-directional hypothesis testing approach; if *t *>*crit*_*upp *_or *t *<*crit*_*low *_, then H_01 _or H_02 _will be rejected for the directional approach, respectively.

**Figure 1:**
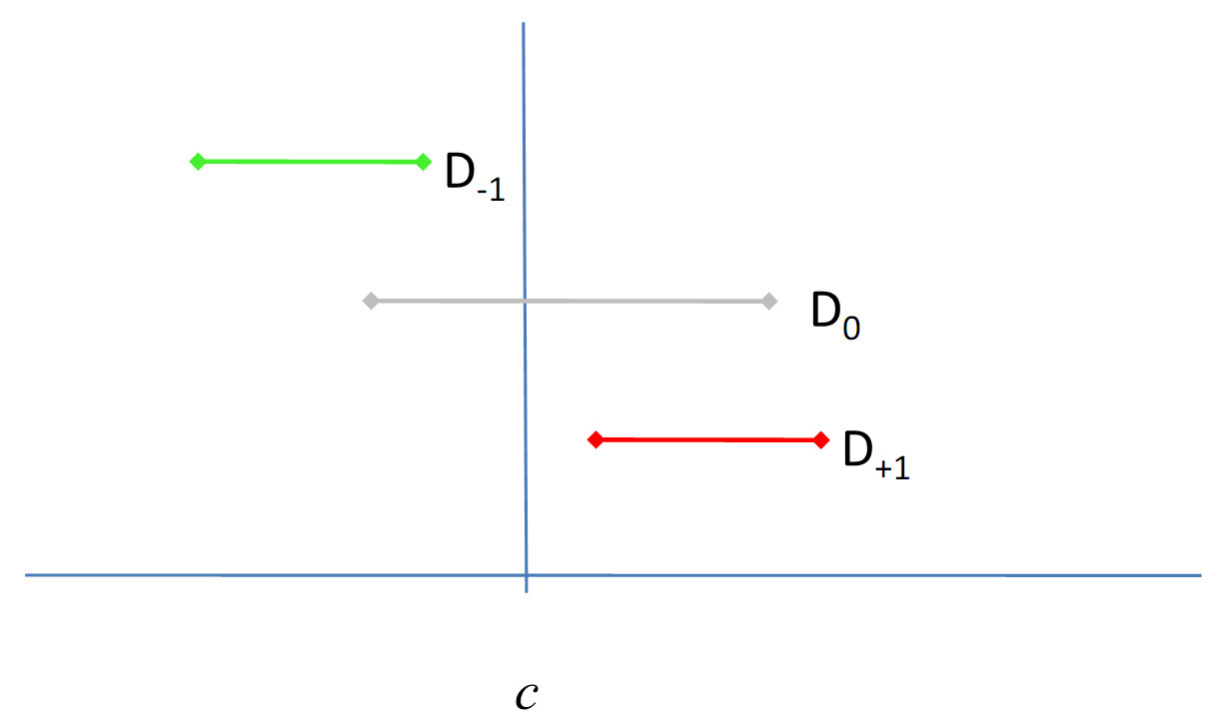
**Schematic representation of possible difference test results depicted with (1-α) confidence intervals**. *D*_-1_, *D*_0 _and *D*_+1 _show a significantly smaller, a not significant and a significantly larger test result, respectively.

The use of a two-sided (1 - α) -confidence interval provides an alternative way to perform a two-sided level α *difference *test. Let  denote the common symmetric (1 - α) -confidence interval for μ. If , then H_0 _will be rejected for the non-directional hypothesis testing approach. If  or , then H_01 _or H_02 _will be rejected for the directional approach, respectively. Three crucial confidence interval scenarios as results of a difference test are depicted in Figure 1. Note that, even if not intended, confidence intervals always provide directional information as well.

### The one-sample t-test as equivalence test

A not significant *difference *test cannot be interpreted as acceptance of the null hypothesis. The population mean μ is only considered *equivalent *to a chosen constant *c *if they do not differ too much, that is, if μ ∈ (*c* - Δ_1_, *c* + Δ_2_). The acceptable differences ∆_1_and ∆_2 _re called equivalence margins and have to be predetermined. Often, ∆_1_=∆_2 _will be chosen for a normally distributed outcome. The equivalence limits *c *- ∆_1 _and *c *+∆_2_form the equivalence range.

Statistical equivalence testing is commonly based on a two one-sided tests (TOST) approach. If both one-sided null hypotheses and  are rejected at a significance level α each, then the population mean μ can be declared *equivalent *to *c*.

The TOST approach can be easily performed by employing a confidence interval. Equivalence will be attained, if the two-sided (1 - 2α)-confidence interval  is completely covered by the equivalence range (*c *- ∆_1,_*c *+ ∆_2_), that is, if  and .

**Figure 2:**
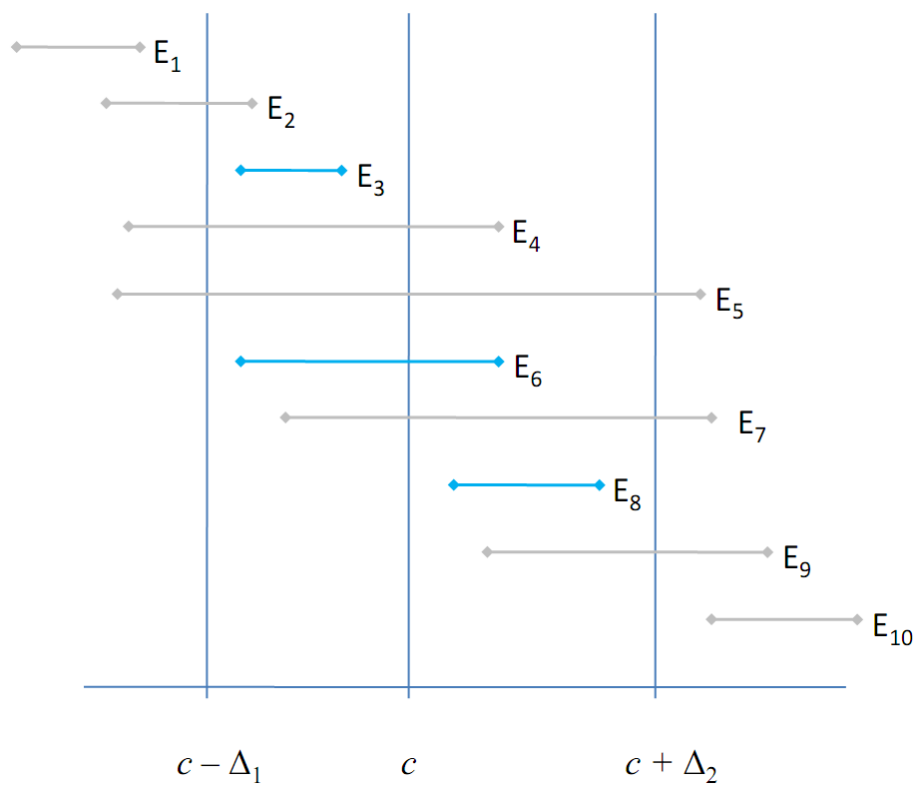
**Schematic representation of possible equivalence test results depicted with (1-2α) confidence intervals**. Scenarios *E*_3_, *E*_6 _and *E*_8 _show equivalence to a chosen constant *c*, whereas all other scenarios show non-equivalence. Note that a (1-2α) equivalence test confidence interval is by definition covered by its corresponding (1-α) difference test confidence interval.

If the equivalence limits *c *- ∆_1 _and *c *+ ∆_2 _together with the null hypothesis value *c *are considered, then ten different equivalence test outcome scenarios can be identified combinatorially (Figure 2). Equivalence would be obtained with scenarios *E*_*3*_, *E*_*6 *_and *E*_*8*_, all other scenarios would be declared not equivalent.

### Combining difference and equivalence tests

If both a difference and an equivalence test are performed on the same sample, then the corresponding (1 - α)-confidence interval of the difference test will cover the (1 - 2α)-confidence interval of the equivalence test. Consequently, provided we have observed a statistically significantly different result, either *D*_-1 _or *D*_+1 _(Figure 1), then in each case three equivalence scenarios, *E*_1 _- *E*_3 _or *E*_8 _- *E*_10_, are possible, respectively (Figure 2). If no significantly different result has been observed (*D*_0_, Figure 1), then any of the ten equivalence scenarios will be conceivable.

This has interesting consequences. If the equivalence interval contains the value *c *which is the case for *E*_4 _- *E*_7_, then the corresponding difference test will show a statistically not significant result. If, however, the equivalence interval does not contain *c *which is the case for *E*_1 _- *E*_3 _and *E*_8 _- *E*_10_, then the result of the difference test will not be immediately evident inasmuch as the difference test confidence interval is at least as wide as its equivalence counterpart.

Admittedly, the scenarios E_1 _| *D*_0 _and E_10 _| *D*_0 _seem implausible, however, they cannot be logically ruled out. Consider, e.g., the combination E_1 _| *D*_0 _which is equivalent to  and . These conditions will only apply, if ∆_1 _and the sample size are sufficiently small and the standard deviation is sufficiently large, respectively.

The combined representation of difference and equivalence test results would have to consider 16 combined scenarios which, however, is a confusing and unfeasible maximal variant. Practically more applicable seems the reduction of the possible equivalence test results to "equivalent" and "not equivalent" which, in combination with the corresponding difference test results, eventually leads to six combined scenarios (Table 1). By considering all equivalent results as one category irrespectively of the difference test result the number of these combinations is reduced to four (Table 1). The category "not equivalent and not significantly different" is a rather uninformative residual category, whereas the other five or three categories contain precise information, respectively.

**Table 1:** Two schemes to distinguish mutual difference and equivalence test results in choropleth maps.

Equivalence test result	Difference test result	Six combined scenarios	Four combined scenarios
*E*_3_	*D*_-1_	equivalent and significantly smaller	equivalent (that is, result of difference test does not matter)
	
*E*_3_, *E*_6_, *E*_8_	*D*_0_	equivalent and not significantly different	
	
*E*_8_	*D*_+1_	equivalent and significantly larger	

*E*_1_, *E*_2_	*D*_-1_	not equivalent and significantly smaller	not equivalent and significantly smaller

*E*_1_, *E*_2_, *E*_4_, *E*_5_, *E*_7_, *E*_9_, *E*_10_	*D*_0_	not equivalent and not significantly different	not equivalent and not significantly different

*E*_9_, *E*_10_	*D*_+1_	not equivalent and significantly larger	not equivalent and significantly larger

Note: The first scheme (column "6 combined scenarios") combines the three difference test results with the two main results of the equivalence test. The second scheme (column "4 combined scenarios") is a simplified alternative to the former. All significantly equivalent results are considered as one category, irrespectively of the result of the difference test. The difference test result only matters then in the case of a not equivalent result.

### Difference and equivalence testing with SMR's

We have motivated difference and equivalence testing with the one-sample t-tests, however, the underlying principle applies to any type of outcome data. E.g., if standardised mortality ratios (SMR's) are considered, then the null hypothesis value *c *will usually be set to one, *c *= 1, and the equivalence limits will be frequently determined by 1 - ∆_1 _= 1/(1 + ∆_2_). A traditional choice in bioequivalence trials will be ∆_1 _= 0.2 [3], which leads to an equivalence range of (0.8, 1.25). Its asymmetry is typical for proportional measurement scales like ratios.

Inferential statistics for SMR's is usually based on the Poisson distribution. In the case of a discrete distribution of the test statistic (e.g. Poisson, Binomial, etc.) the computation of p-values and confidence intervals can be performed with the so-called twice-the-smaller-tail (TST) method ([4], p. 59). That is, the two-sided test results at level α are derived from a combination of the two corresponding one-sided results at level α/2 each ([4], p. 60). Obviously, equivalence testing by TOST is TST per definition. However, the TST method can become rather conservative [4].

Our method requires that a (1 - α)-confidence interval covers its corresponding (1 - 2α)-confidence interval. This so-called property of nestedness [4] seems to be naturally met in general, however, it is not guaranteed in the field of discrete data and permutation tests, when different confidence interval construction principles are applied. That is, a non-TST (1 - α)-confidence interval of the difference test does not necessarily cover the TST (1 - 2α)-confidence interval of the corresponding equivalence test [4]. The property of nestedness may also become an issue if the conservativeness of the TST method is reduced by employing the so-called mid-p correction [4].

### Multiple testing

Jointly performing a difference and an equivalence test for a single spatial unit maintains the multiple level of significance at α [for a proof see Additional file 1].

Performing such combined tests for a multitude of spatial units inevitably increases the risk for type I and type III (directional) errors. Adjustments for multiple testing can be applied as long as the property of nestedness [4] is maintained, that is, as long as the multiplicity-adjusted confidence interval of a difference test still covers the multiplicity-adjusted confidence interval of the corresponding equivalence test.

### The Bayesian approach

Wellek [5] notices that in situations, where Bayesian credible intervals coincide with classical confidence intervals, a Bayesian equivalence testing procedure in analogy to the classical TOST approach can be applied. We propose - analogous to the described classical approach - to combine (1 - α)- and (1 - 2α)-credibility intervals to a sort of combined Bayesian difference and equivalence testing approach.

The specified prior distribution and the observed data are used to determine the posterior distribution of the parameter of interest, which is considered a random variable then. A Bayesian difference test can now be derived from the posterior probability that the parameter of interest exceeds the value *c*. The posterior probability that the parameter lies within the equivalence range (*c *- ∆_1_,*c *+ ∆_2_) provides the basis of Bayesian equivalence decision-making.

## Results

**Figure 3:**
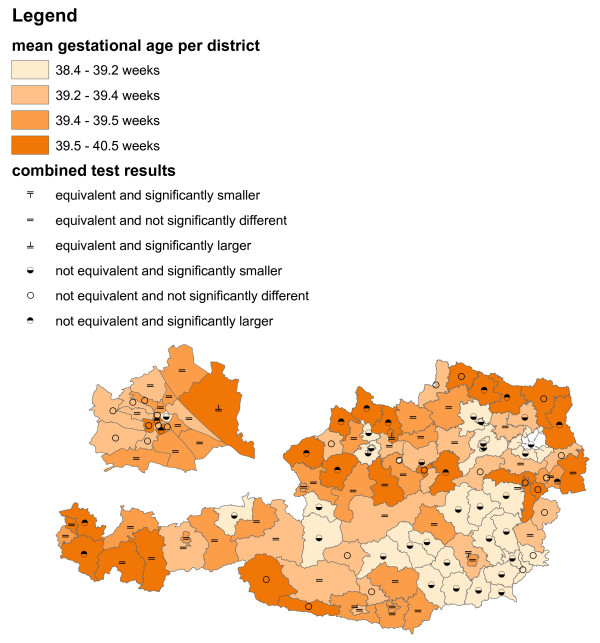
**Gestational age in Austria 2008**. Different colours refer to different mean gestational ages (in weeks), different symbols refer to different results of a difference/equivalence test combination ("6 combined scenarios").

**Figure 4:**
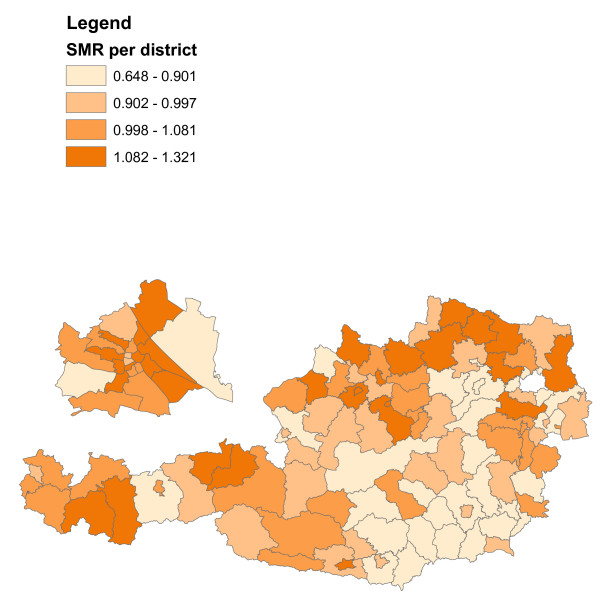
**SMR's of infant mortality in Austria 1984-2007**. Different colours refer to different SMR's.

**Figure 5:**
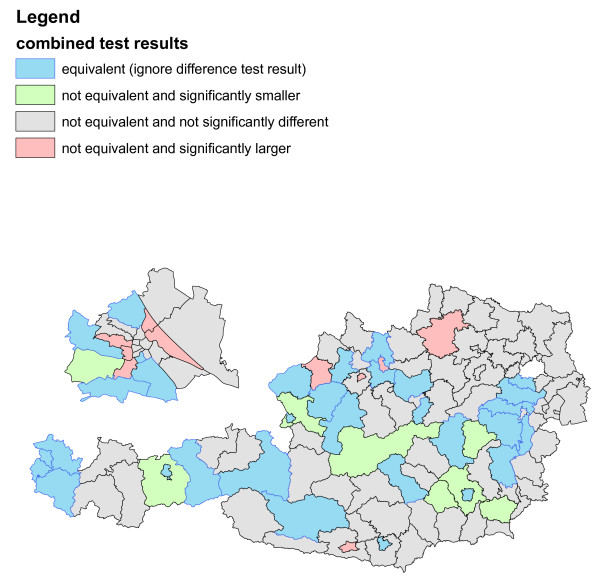
**Difference/equivalence test results for infant mortality in Austria 1984-2007**. Different colours refer to different results of a difference/equivalence test combination ("4 combined scenarios").

The following two examples are based on Austrian vital statistics data (source: Statistics Austria [6]) which includes all births in Austria from 1970 onwards. The Republic of Austria consists of 121 administrative districts, from which 23 (19%) constitute the densely populated capital city Vienna. About 1.7 million (20%) out of about 8.4 million inhabitants live in Vienna. For the sake of better visualisation we have cut out Vienna in our choropleth maps from its location in the north-east of Austria, magnified it and placed it above the western districts (Figures 3, 4 and 5). The choropleth maps have been produced with ArcGIS 9. A significance level α = 0.05, as is customary in medicine, was used throughout the examples.

### Gestational age in Austria 2008

In 2008, a total of 60,303 newborns with Austrian mothers had been recorded from where we analysed gestational age within the administrative districts. Tests for equivalence and difference were done in SAS using the procedure TTEST (option TOST for equivalence test). The equivalence range (*c *- ∆_1,_*c *+ ∆_2_) was set to a width of 4 days, i.e. ∆_1 _= ∆_2 _= 2/7 = 0.286 weeks and *c *was set to the sample mean  of Austrian newborns recorded from 1999 to 2007, that is,  weeks.

The mean gestational ages of the districts are split at the quartiles into four categories which are represented with different colours. Six combined scenarios of the equivalence/difference test results are represented with different symbols (Table 1). Both, colours and symbols are displayed together in one graphic (Figure 3).

The non-random spatial distribution of mean gestational ages is obvious (Figure 3). In particular, shorter gestational age seems to be common in the south-eastern parts of Austria. Prolonged gestational age becomes more frequent in the north-eastern, central and western parts of Austria. The equivalence/difference testing information supports this impression (Figure 3). There is an equivalence/difference testing symbol in the opening which appeared after cutting out the capital Vienna. It belongs to a district surrounding Vienna which consists of several spatially separated areas.

Jointly displaying the variable of interest with colours and the equivalence/difference test results with symbols clearly emphasizes the former over the latter. Detailed information for specific districts can be retained on closer inspection, but it is rather difficult to get an overall spatial impression of the equivalence/difference testing information from this form of graphical representation.

### Infant mortality in Austria 1984-2007

Infant mortality (death of a live birth during the first year) was recorded between 1984 and 2007 in 121 administrative districts. A total of 10,914 out of 1,985,203 live births deceased. Inclusion criteria for the data set were that the infants had been born as singletons between the 24th and 44th week of gestation to mothers between 13 to 50 years of age. Expected numbers of cases per district were calculated by multiplying the national infant mortality rate with district specific numbers of births. Standardized mortality ratios were calculated as in Waldhoer et al. [7]. Equivalence and difference tests were performed with the SAS macro of Daly [8]. The equivalence range (0.8, 1.25) was used.

The district SMR's are split at the quartiles into four categories and represented with different colours (Figure 4). Four combined scenarios of the equivalence/difference test results are as well represented with different colours in a separate graphic (Table 1, Figure 5).

The results of the combined equivalence/difference test results in Figure 5 only partly confirm the results of Figure 4 as more than half of the districts do not allow conclusive decisions.

Separately representing the variable of interest and the equivalence/difference test results with colours puts equal emphasis on both features which is in contrast to the colours/symbols representation of Figure 3.

## Discussion and conclusions

The two examples (Figure 3 and Figures 4-5) are prototypic for the two different main motivations of integrating difference and equivalence test results into choropleth maps. The main aim of Figure 3 is a concise combination of the spatial distribution of the variable of interest and the statistical test results, where the focus is on the former and the latter is meant to provide supplementary information only. On the other hand, Figures 4 and 5 show a situation where both, data description and statistical testing are of equal interest. It should be noted that other forms of graphical representation could be considered to effectively communicate the bivariate information [see e.g. [9-11]].

The non-random spatial distribution of infant mortality in Figure 4 closely resembles that of gestational age in Figure 3. Shorter gestational age seems to be associated with decreased infant mortality. Drawing causal relationships, however, may be fallacious for two reasons. Firstly, the study times do not overlap (2008 in Figure 3 and 1984-2007 in Figure 4), and secondly, there is the possibility of an ecological inference fallacy.

Figure 4 provides a rather typical example of a traditional epidemiological choropleth map. The clear non-random spatial distribution in Figure 4 exhibits many districts with increased and decreased risk in the north-west and south-east of Austria, respectively. When defining a range from 0.8 to 1.25 as equivalent and therefore not important enough to raise public health concerns, then about 40% of the districts will allow conclusive decisions (red-, green- and blue-coloured districts in Figure 5). The indefiniteness of the gray-coloured districts may be mainly due to lack of statistical power, however, in any case valuable additional information for local health authorities is provided.

Both examples differ in a further small, but crucial detail. In the gestational age example, the null hypothesis value *c *is determined from a previous data set (1999 to 2007) in order to test the various districts in the data set at hand (2008), and both data sets do not overlap.

In the infant mortality example, on the contrary, all live births from 1984 to 2007 were used to calculate the national infant mortality rate which, via SMR, is tested against the district infant mortality rates from 1984 to 2007. That is, the null hypothesis is partially determined by the data which are to be tested. This means that a districts rate is compared with all other district rates including its own one. Although such an approach is statistically questionable, it is quite common in spatial epidemiology. As long as the number of cases and the size of the population of the respective district are small compared to the whole national sample, the thereby arising bias can be safely ignored. Note that there is a structurally similar problem in the field of relative survival where people suffering from an illness are compared to the overall population including the diseased ones.

Both examples are based on fixed effect estimators, which neither do account for spatial autocorrelation in the underlying variables nor do correct for the inherent multiplicity. It would be rather straightforward to translate the approach to random or mixed effect models with either global or local shrinkage (spatial smoothing) which "borrow strength" from adjacent districts. Corrections for multiple testing could be performed within the models in order to account for reduced degrees of freedom by positive spatial autocorrelation. Multiple testing will not be an explicit issue if spatial smoothing is performed within a Bayesian setting as "a correct adjustment is automatic within the Bayesian paradigm" [12].

Note that the decision for a multiplicity adjustment before reporting public health results has to consider both technical and non-technical points. Multiplicity is an issue to be kept in mind when looking from a nationwide or transregional level at a series of regional test results. On the contrary, individual persons and local health authorities may only be interested in their corresponding local area results. Similar arguments apply for the choice between simple fixed effect and spatially smoothed estimates. There might be local health authorities, who might resist against a seeming degradation of their spotless public health records by the inclusion of ill-performing neighboring districts due to spatial smoothing. Critics from the local residents and the media might argue that shrinkage and smoothing is merely a convenient tool for understating unpleasant results, particularly, as spatial smoothing may yield essentially conservative results [13]. On the other hand, spatially smoothed results are more stable and less prone to random fluctuations. Governmental agencies interested in an overall picture may favor them.

Concluding, we think that enhancing spatial maps with a combination of statistical difference and equivalence test results could help to classify epidemiological findings the right way. A better understanding of spatial observations could be achieved by explicitly defining their relevance through a pre-defined equivalence range. In order to apply our suggested method all is needed are confidence intervals or - in a Bayesian setting - credibility intervals for the small area parameters of interests. Technically, it does not matter whether or not these intervals have been "preprocessed" by multiplicity adjustments or spatial smoothing.

Finally note that *equivalence *and *difference *may have other sensible meanings than those employed here. In the field of spatial epidemiology *equivalence *may be considered as spatial clustering and *difference *may be related to spatial outliers or excessive observations. Examples of methods which address these notions of *equivalence *and *difference *in combination include LISA statistics [14] and Oden's *I*_*pop *_[15].

## Competing interests

The authors declare that they have no competing interests.

## Authors' contributions

Both authors contributed equally to this research. Both authors read and approved the final manuscript.

## Supplementary Material

Additional file 1 **Does the joint application of a difference and an equivalence test pose a multiple testing problem**? It is stated in the Multiple testing subsection that jointly performing a difference and an equivalence test for a single spatial unit maintains the multiple level of significance at α. A formal proof for this statement is provided. Click here for file

## References

[B1] Sonnemann E Allgemeine Lösungen multipler Testprobleme EDV in Medizin und Biologie 1982 13 4 120 128 English version of the original article with minor corrections by Finner H: **General Solutions to Multiple Testing Problems**. Biometrical Journal 2008, **50**(5):641-656. 10.1002/bimj.200810462 18932150

[B2] Hauschke D Steinijans VW Directional decision for a two-tailed alternative Letter to the editor. Journal of Biopharmaceutical Statistics 1996 6 2 211 213 10.1080/10543409608835134 8732915

[B3] Food and Drug Administration Guidance for Industry. Bioavailability and Bioequivalence Studies for Orally Administered Drug Products - General Considerations 2003 http://www.fda.gov/downloads/Drugs/GuidanceComplianceRegulatoryInformation/Guidances/ucm070124.pdf Revision 1

[B4] Hirji KF Exact analysis of discrete data 2006 Boca Raton: Chapman & Hall/CRC ISBN-13: 978-1584880707

[B5] Wellek S Testing Statistical Hypotheses of Equivalence 2003 Boca Raton: Chapman & Hall/CRC ISBN-13: 978-1584881605

[B6] Austria Statistics Vital statistics http://www.statistik.at/web_en/statistics/population/births/index.html

[B7] Waldhoer T Wald M Heinzl H Analysis of the spatial distribution of infant mortality by cause of death in Austria in 1984 to 2006 International Journal of Health Geographics 2008 7 21 10.1186/1476-072X-7-21 18495006 PMC2432051

[B8] Daly L Simple SAS macros for the calculation of exact binomial and Poisson confidence limits Computers in Biology and Medicine 1992 22 5 351 361 10.1016/0010-4825(92)90023-G 1424580

[B9] Carr DB Wallin JF Carr DA Two new templates for epidemiology applications: linked micromap plots and conditioned choropleth maps Statistics in Medicine 2000 19 17-18 2521 2538 10.1002/1097-0258(20000915/30)19:17/18<2521::AID-SIM585>3.0.CO;2-K 10960869

[B10] Bell BS Hoskins RE Pickle LW Wartenberg D Current practices in spatial analysis of cancer data: mapping health statistics to inform policymakers and the public International Journal of Health Geographics 2006 5 49 10.1186/1476-072X-5-49 17092353 PMC1647272

[B11] Pickle LW Carr DB Visualizing health data with micromaps Spatial and Spatio-temporal Epidemiology 2010 1 2-3 143 150 10.1016/j.sste.2010.03.007 22749470

[B12] Bayarri MJ Berger JO The Interplay of Bayesian and Frequentist Analysis Statistical Science 2004 19 1 58 80 10.1214/088342304000000116

[B13] Richardson S Thomson A Best N Elliott P Interpreting Posterior Relative Risk Estimates in Disease-Mapping Studies Environmental Health Perspectives 2004 112 9 1016 1025 10.1289/ehp.6740 15198922 PMC1247195

[B14] Anselin L Local Indicators of Spatial Association--LISA Geographical Analysis 1995 27 2 93 115 10.1111/j.1538-4632.1995.tb00338.x

[B15] Oden N Adjusting Moran's I for population density Statistics in Medicine 1995 14 1 17 26 10.1002/sim.4780140104 7701154

