# Supplementary material for: Combining difference and equivalence test results in spatial maps
Source: Int J Health Geogr. 2011 Jan 10;10:3. doi: 10.1186/1476-072X-10-3 (PMC3032638; doi:10.1186/1476-072X-10-3)
Supplement: Additional file 1 — Does the joint application of a difference and an equivalence test pose a multiple testing problem? It is stated in the Multiple testing subsection that jointly performing a difference and an equivalence test for a single spatial unit maintains the multiple level of significance at α. A formal proof for this statement is provided. [file 1476-072X-10-3-S1.DOC]

Additional material for the manuscript “*Combining difference and equivalence test results in spatial maps*” by Thomas Waldhoer and Harald Heinzl:

**Does the joint application of a difference and an equivalence test pose a multiple testing problem?**

In the following, μ will be used as generic symbol. It is not restricted to a population mean as in the manuscript.

Case differentiation:

- Case A: $\mu\in\left(-\infty,c-\Delta_{1}\right]$
- Case B: $\mu\in\left(c-\Delta_{1},c\right)$
- Case C: $\mu=c$
- Case D: $\mu\in\left(c,c+\Delta_{2}\right)$
- Case E: $\mu\in\left[c+\Delta_{2},\infty\right)$

Note that case A is laterally reversed to case E, and case B is laterally reversed to case D. Hence, only cases A-C will be considered in the following.

The 16 possible combinations of equivalence and difference test results have been pooled in the paper (see Table 1) into

- six combined scenarios
- four combined scenarios

The possible type I and type III (directional) errors for each case and each scheme of combined scenarios are specified and proofs are given that the error probabilities are smaller-equal to the chosen significance level α.

Case A, six combined scenarios: $\mu\in\left(-\infty,c-\Delta_{1}\right]$

The sum of the probabilities of all three “equivalent” scenarios and the “not equivalent and significantly larger” scenario should be smaller-equal to α, that is:

$$P\left(\left[\bar{x}_{low,\alpha}>c-\Delta_{1}\right]\wedge\left[\bar{x}_{upp,1-\alpha}<c+\Delta_{2}\right]\right)+P\left(\left[\bar{x}_{low,\alpha/2}>c\right]\wedge\left[\bar{x}_{upp,1-\alpha}\geq c+\Delta_{2}\right]\right)\leq\alpha$$

Proof of case A, six combined scenarios:

$$\begin{matrix} P\left(\left[\bar{x}_{low,\alpha}>c-\Delta_{1}\right]\wedge\left[\bar{x}_{upp,1-\alpha}<c+\Delta_{2}\right]\right)+P\left(\left[\bar{x}_{low,\alpha/2}>c\right]\wedge\left[\bar{x}_{upp,1-\alpha}\geq c+\Delta_{2}\right]\right)\leq\\ P\left(\left[\bar{x}_{low,\alpha}>c-\Delta_{1}\right]\wedge\left[\bar{x}_{upp,1-\alpha}<c+\Delta_{2}\right]\right)+P\left(\left[\bar{x}_{low,\alpha}>c-\Delta_{1}\right]\wedge\left[\bar{x}_{upp,1-\alpha}\geq c+\Delta_{2}\right]\right)= \\ P\left(\bar{x}_{low,\alpha}>c-\Delta_{1}\right)\leq\alpha\end{matrix}$$

This is true per definition as $\mu\in\left(-\infty,c-\Delta_{1}\right]$ in this case.

Case B, six combined scenarios: $\mu\in\left(c-\Delta_{1},c\right)$

The sum of the probabilities of both “significantly larger” scenarios should be smaller-equal to α, that is:

$$P\left(\bar{x}_{low,\alpha/2}>c\right)\leq\alpha$$

Proof of case B, six combined scenarios:

This is true per definition as $\mu\in\left(c-\Delta_{1},c\right)$ in this case.

Case C, six combined scenarios: $\mu=c$

The sum of the probabilities of all four “significantly different” scenarios should be smaller-equal to α, that is:

$$P\left(\bar{x}_{upp,1-\alpha/2}<c\right)+P\left(\bar{x}_{low,\alpha/2}>c\right)\leq\alpha$$

Proof of case C, six combined scenarios:

This is true per definition as $\mu=c$ in this case.

Case A, four combined scenarios: $\mu\in\left(-\infty,c-\Delta_{1}\right]$

The sum of the probabilities of the “equivalent” scenario and the “not equivalent and significantly larger” scenario should be smaller-equal to α, that is:

$$P\left(\left[\bar{x}_{low,\alpha}>c-\Delta_{1}\right]\wedge\left[\bar{x}_{upp,1-\alpha}<c+\Delta_{2}\right]\right)+P\left(\left[\bar{x}_{low,\alpha/2}>c\right]\wedge\left[\bar{x}_{upp,1-\alpha}\geq c+\Delta_{2}\right]\right)\leq\alpha$$

Proof of case A, four combined scenarios:

This is the same as for case A, six combined scenarios.

Case B, four combined scenarios: $\mu\in\left(c-\Delta_{1},c\right)$

The probability of the “not equivalent and significantly larger” scenario should be smaller-equal to α, that is:

$$P\left(\left[\bar{x}_{low,\alpha/2}>c\right]\wedge\left[\bar{x}_{upp,1-\alpha}\geq c+\Delta_{2}\right]\right)\leq\alpha$$

Proof of case B, four combined scenarios:

$$\begin{matrix} P\left(\left[\bar{x}_{low,\alpha/2}>c\right]\wedge\left[\bar{x}_{upp,1-\alpha}\geq c+\Delta_{2}\right]\right)\leq\\ P\left(\bar{x}_{low,\alpha/2}>c\right)\leq\alpha\end{matrix}$$

This is true per definition as $\mu\in\left(c-\Delta_{1},c\right)$ in this case.

Case C, four combined scenarios: $\mu=c$

The sum of the probabilities of the “not equivalent and significantly smaller” and the “not equivalent and significantly larger” scenario should be smaller-equal to α, that is:

$$P\left(\left[\bar{x}_{upp,1-\alpha/2}<c\right]\wedge\left[\bar{x}_{low,\alpha}\leq c-\Delta_{1}\right]\right)+P\left(\left[\bar{x}_{low,\alpha/2}>c\right]\wedge\left[\bar{x}_{upp,1-\alpha}\geq c+\Delta_{2}\right]\right)\leq\alpha$$

Proof of case C, four combined scenarios:

$$\begin{matrix} P\left(\left[\bar{x}_{upp,1-\alpha/2}<c\right]\wedge\left[\bar{x}_{low,\alpha}\leq c-\Delta_{1}\right]\right)+P\left(\left[\bar{x}_{low,\alpha/2}>c\right]\wedge\left[\bar{x}_{upp,1-\alpha}\geq c+\Delta_{2}\right]\right)\leq\\ P\left(\bar{x}_{upp,1-\alpha/2}<c\right)+P\left(\bar{x}_{low,\alpha/2}>c\right)\leq\alpha\end{matrix}$$

This is true per definition as $\mu=c$ in this case.
